# Association Between Maternal Obesity and Cesarean Delivery Complications

**DOI:** 10.7759/cureus.7163

**Published:** 2020-03-02

**Authors:** Zaheera Saadia

**Affiliations:** 1 Obstetrics and Gynecology, Qassim University College of Medicine, Buraidah, SAU

**Keywords:** body mass index, caesarean section, complications, obesity, pregnancy outcomes

## Abstract

Background

Several studies suggest that maternal obesity might be associated with intraoperative and postoperative complications of cesarean delivery. However, these results are not validated in the Pakistani population.

Aim

We aimed to assess the association between maternal obesity and intraoperative and postoperative complications of cesarean delivery.

Methods

We performed a retrospective observational study recording the prevalence of intraoperative and postoperative complications in women undergoing cesarean delivery. For all consecutive cesarean deliveries in Fehmida Sarfaraz hospital, Sialkot, Pakistan, we recorded the data of the maternal age, weight, body mass index (BMI), gestational age at delivery, intraoperative and postoperative complications, and the adverse pregnancy outcomes. We used the chi-square test, Spearman correlation, and linear regression to test the relationship between the study variables.

Results

We included 245 women in this study (non-obese group: n = 83; obese group: n = 162). BMI positively correlated with the incidence of deep venous thrombosis (DVT; r = 0.249), endometritis (r = 0.148), pyrexia (r=0.139), and wound infections (r = 0.155). Also, BMI could significantly predict DVT (Beta coefficient 2.886, P = 0.003), hospital stay (Beta coefficient 0.801, P = 0.001), pyrexia (Beta coefficient 0.819, P = 0.003), and wound infection (Beta coefficient 0.449, P = 0.049).

Conclusion

Our data suggest that BMI was significantly correlated with several cesarean section (CS) delivery complications. Obese women undergoing CS delivery are at higher risk of several CS delivery complications. Also, they had a longer hospital stay and higher birth weight for their neonates compared with non-obese women. Future multicentre studies are needed in our population to determine the magnitude of risk across different BMI subgroups.

## Introduction

About 830 women die every day from complications of pregnancy and childbirth. Most of them (99%) are in developing countries. The rates of childbirth complications are increasing worldwide. For example, the childbirth complications in the US has increased by 45% over the last decade [1]. The World Health Organization estimates that about 14 million women suffer from postpartum hemorrhage every year.

The literature suggests that pre-pregnancy obesity might have a negative impact on pregnancy outcomes and childbirth complications. Some reports suggested a correlation between high maternal BMI and hypertensive disorders, fertility, cesarean section (CS), maternal mortality, neonatal Apgar score, neonatal admission to the neonatal intensive care unit (NICU), preterm delivery, congenital defects, birth weight, weight status after birth, child morbidity, and respiratory problems as asthma and children's mortality [2]. However, the clear relationship between maternal BMI and these outcomes have not been clarified yet. Also, most of these data come from developed countries where obesity is said to be more prevalent; however, obesity is getting a problem of underdeveloped countries as well because of lack of awareness.

In terms of cesarean delivery, a large body of evidence supports a positive association between pre-pregnancy BMI and cesarean delivery which makes women with higher BMI at a greater risk of delivery complications compared to those with lower BMI [3-6]. Also, women with higher BMI were found to have more time from decision till delivery and more epidural anesthesia failures compared to those with non-obese [7].

It was reported that excess gestational weight gain, gestational diabetes, hypertensive complications, cesarean delivery, and increased infant weight were independently associated with pre-pregnancy obesity [8].

Women with gestational diabetes have an increased risk of pregnancy complications; however, the risks are more in obese women with obese [9]. Another study by McPherson *et al*. showed an increased risk of adverse delivery outcomes in the subgroup of morbidly obese women [10].

Several studies suggest that maternal obesity might be associated with intraoperative and postoperative complications of cesarean delivery. However, these results are not validated in the Pakistani population. Therefore, we conducted this prospective study to evaluate whether higher BMI was associated with more adverse pregnancy outcomes and childbirth complications in the Pakistani population.

## Materials and methods

We followed the “Strengthening the Reporting of Observational Studies in Epidemiology” (STROBE) statement guidelines when reporting this manuscript [11]. The study was approved by the ethics committee in Fehmida Sarfaraz hospital, Sialkot, Pakistan.

Study design, setting, and duration

We conducted a retrospective observational study in the department of obstetrics at the Fehmida Sarfaraz Hospital, Pakistan. The study population is defined as pregnant women attending the study center within the period from January to December 2018. All pregnant women undergoing their first CS delivery were eligible for inclusion in the study. We excluded cases of normal labor, premature rupture of membranes, any woman suffering from any medical condition not related to pregnancy, and those with previous CS.

Source of data and the study variables

For each case, we recorded the following data: (1) demographics, (2) type of placenta, (3) gestational history, (4) weight, height, and BMI, (5) indication of CS, (6) postpartum hemorrhage, blood loss, and the amount of transfused blood units, (7) NICU and hospital stays, (8) injuries, (9) congenital anomalies, and (10) maternal and neonatal complications. Data were entered in a self-structured proforma and were later transferred to SPSS for analysis. Height and Weight Scale DETECTO 339 was used to measure height and weight.

Statistical analysis

Categorical data were summarized as frequencies and percentages, while continuous data were presented as mean and standard deviation. For comparison of categorical and continuous variables, we used the chi-square test and Student's t-test, respectively. The point-biserial correlation was calculated to estimate the direction and magnitude of the correlation between BMI and the delivery outcomes represented as dichotomous data. The Pearson correlation was used to investigate the correlations between continuous variables. To investigate whether BMI could predict the delivery outcomes, we constructed multiple binary logistic regression models. An alpha level below 0.05 was considered for statistical significance. All analyses were done using SPSS statistical software (version 25, for windows).

## Results

Characteristics of the study population

Our study included 245 women with an average height of 153 (±4.9) cm, an average weight of 76.5 (±14.3) kg, and an average BMI of 32.7 (±6.2) kg/m^2^. The average duration of pregnancy was 37.5 (±2) weeks, and the average birth weight of their neonates was 3.4 (±2.3) kg. The two BMI groups of our study included 83 women for the non-obese group (BMI ˂30 kg/m^2^) and 162 women for the obese group (BMI ≥30 kg/m^2^).

There were no cases of maternal mortality, pulmonary embolism, or birth traumas. Therefore, these three variables were omitted from the comparison, correlation, and regression analyses. The demographic characteristics of the two subgroups are shown in Table 1.

**Table 1:** A summary of the characteristics of the two study groups BMI, body mass index; NICU, neonatal intensive care unit; HB, hemoglobin

Variable		Non-obese group (n=83)	Obese group (n=162)	P value
Age (years)		33.2 (± 5.8)	33.3 (± 5.5)	0.904
Height (cm)		153.9 (± 5.1)	152.5 (± 4.8)	0.041
Weight (kg)		60.9 (± 7.5)	84.5 (± 9.6)	˂0.001
BMI (kg/m^2^)		25.8 (± 3)	36.3 (± 4.1)	˂0.001
Duration of pregnancy (weeks)		37.3 (± 2.3)	37.6 (± 1.8)	0.244
BMI ranges	Below-25	32 (38.6%)	0 (0%)	˂0.001
	25-29.9 overweight	51 (61.4%)	0 (0%)	
	30-34.9 obese level 1	0 (0%)	74 (45.7%)	
	35-39.9 obese level 2	0 (0%)	51 (31.5%)	
	40 and above severely obese	0 (0%)	37 (22.8%)	

The indication of CS in the two study groups

**Figure 1:**
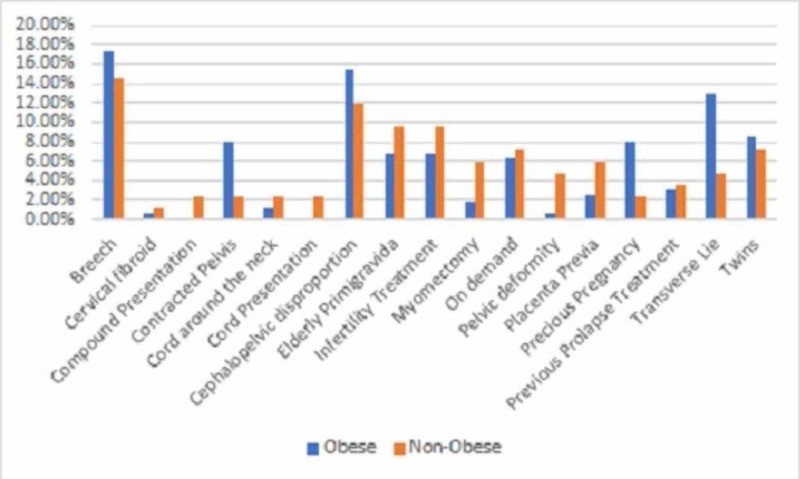
The percentages of the cesarean section indication in the study groups

In the non-obese group, the most common indications for CS were breech (14.5%), CPD (12%), elderly PG (10.8%), and infertility TM (9.6%). In the obese group, the most common indications for CS were breech (17.3%), CPD (15.4%), and transverse lie (13%). The percentages of the CS indications of the two groups are shown in Figure 1.

Correlation between BMI and pregnancy outcomes

The correlation analysis showed that BMI was significantly correlated with birth weight (r = 0.118), deep venous thrombosis (DVT; r = 0.249), endometritis (r = 0.148), hospital stay (r = 0.163), preterm (r = -0.142), pyrexia (r = 0.139), and wound infection (r = 0.155). The coefficients of the correlation between BMI and the delivery outcomes are shown in Table 2.

**Table 2:** The correlation between BMI and the delivery outcomes BMI, body mass index; NICU, neonatal intensive care unit; Hb, hemoglobin

	Correlation Coefficient	P-value
Age	0.039	0.374
APGAR score	-0.076	0.134
Birth trauma	0.068	0.193
Birth weight	0.118	0.008
Blood loss	0.009	0.87
Cesarean hysterectomy	0.068	0.196
Chest infection	0.049	0.351
Congenital malformation	-0.078	0.135
Deep venous thrombosis	0.249	˂0.0005
Duration of pregnancy	0.024	0.595
Endometritis	0.148	0.005
Hb Fall	0.083	0.065
Hospital stay	0.163	0.001
Intraoperative injury	0	1
NICU stay	0.018	0.726
Post-partum hemorrhage	0.062	0.238
Preterm	-0.142	0.007
Pyrexia	0.139	0.008
Units of blood transfusion	-0.002	0.972
Urinary tract infection	-0.042	0.429
Wound infection	0.155	0.003

Regression models of BMI to predict pregnancy outcomes

The regression analysis showed that BMI could significantly predict DVT (Beta coefficient 2.886, P = 0.003), hospital stay (Beta coefficient 0.801, P = 0.001), pyrexia (Beta coefficient 0.819, P = 0.003), and wound infection (Beta coefficient 0.449, P = 0.049). However, BMI could not significantly predict birth weight or endometritis. The results of the regression analysis models are shown in Table 3.

**Table 3:** Regression analysis results between BMI and the delivery outcomes BMI, body mass index

	Beta regression coefficient	P-value
Birth weight	0.016	0.029
Deep venous thrombosis	2.886	0.003
Endometritis	16.949	0.994
Hospital stay	0.801	0.001
Preterm	-0.613	0.029
Pyrexia	0.819	0.003
Wound infection	0.449	0.049

.

## Discussion

This study showed that obese women (BMI≥30 kg/m^2^) had more incidence of DVT, pyrexia, wound infection, endometritis, and longer hospital stays and higher birth weight of their neonates compared to non-obese women (BMI ˂30 kg/m^2^). The correlation analysis showed that BMI positively correlated with the DVT, pyrexia, wound infection, endometritis, birth weight, and hospital stays. The regression analysis showed that BMI could significantly predict DVT, endometritis, hospital stay, preterm, pyrexia, wound infection.

Study findings and comparisons

The higher risk of DVT in obese women could be explained in that obesity is thrombogenic. Literature showed that obesity was associated with thrombogenic events in men and women population [12]. Also, obese individuals are likely to exert less mobility and less physical activity which makes them more susceptible to thromboembolic events. Moreover, the results of this study might have been influenced by the pattern of obesity in the study region. Therefore, the non-obese group included several women with a relatively obese that would classify them as “overweight.”

Nonetheless, our study expands the literature by highlighting several complications and delivery outcomes that are significantly correlating with BMI. DVT, pyrexia, wound infection, endometritis, birth weight, and hospital stays were positively correlated with pre-pregnancy BMI. These positive correlations were statistically significant in our population which opens the door for further evaluation about the relationship between BMI and CS delivery outcomes.

Several studies in the literature reported the association between BMI and delivery complications. Durnea *et al*. analyzed data from 45,557 deliveries and they found that increased BMI was associated with a reduced risk of minor perineal trauma [13]. A large analysis of 51,218 women data with different BMIs showed that women with BMI ranging from 40 to 49.9 kg/m^2^ had a lower risk of any intraoperative complications compared with less obese women with BMI ranging from 18.5 to 29.9 kg/m^2^ (RR 0.76, 95% CI [0.64 to 0.89]). This could be explained as an effect modification by more super-obese women undergoing emergency CS to avoid the expected complications [14]. When maternal obesity is associated with gestational diabetes, the risk of maternal morbidity and delivery complications was higher compared to those with obesity only without gestational diabetes and those with gestational diabetes only without obesity. This suggests that the combined effect of obesity and gestational diabetes might increase the risk of maternal and fetal complications [14].

In terms of the financial burden, the costs of childbirth for obese women were significantly higher than those for non-obese women, which reflects the impact of more CS, more preterm deliveries, and the longer hospitalization period among obese women [15].

The literature suggests that the impact of pre-pregnancy obesity extends more beyond childbirth complications. A meta-analysis of published studies showed that children born to obese mothers have higher odds of compromised neurodevelopmental outcomes [16]. Also, pre-pregnancy obesity increased the risks of attention-deficit hyperactivity disorder, autism spectrum disorder, developmental delay, and emotional/behavioral problems [16].

The lack of correlation between the other CS delivery complications and BMI in this study could be explained by effect modification in clinical practice. Obstetricians are more cautious with the group of obese women during CS since obese women have been widely regarded in the literature as a high-risk group during CS. The prophylactic measures in our center might have affected the risk assessment. Nonetheless, our study is consistent with the previous literature suggesting that pre-pregnancy BMI is correlated with more CS complications. Therefore, obese women should consider effective weight reduction methods before pregnancy to avoid possible complications and morbidity.

Strengths and limitations

The strengths of our study are: (1) we conducted a prospective observational study to ensure including all relevant complications and avoid missing any complications from the historical records, and (2) our study is conducted in a region where obesity is prevalent compared to other regions. Nonetheless, our study is limited by the inclusion of overweight women in the control group owing to the relatively small sample size. To overcome the obesity pattern in the underlying population, future multicentre collaboration is recommended to recruit a larger number of women across a wide range of BMI.

## Conclusions

Our data suggest that BMI was significantly correlated with several CS delivery complications. Obese women undergoing CS delivery are at a higher risk of several CS delivery complications such as DVT, pyrexia, wound infection, and endometritis. Besides, they had a longer hospital stay and higher birth weight for their neonates compared with non-obese women. Future multicentre studies are needed in our population to determine the magnitude of risk across different BMI subgroups. Therefore, obesity in pregnancy is not only a problem in developed countries but also in developing countries and it is time to concentrate on running awareness programs from now to prevent the problems in the future.
